# Supplementary material for: Stable isotope analysis of multiple tissues from Hawaiian honeycreepers indicates elevational movement
Source: PLoS One. 2020 Jul 15;15(7):e0235752. doi: 10.1371/journal.pone.0235752 (PMC7363098; doi:10.1371/journal.pone.0235752)
Supplement: Attachment — Submitted filename: Comments.docx [file pone.0235752.s001.docx]

Grammatical Comments:

Line 38: In the abstract I believe you use the word “where” instead of “were”

Lines 201-202: You used the word “powered” instead of “powdered” twice.

Specific Comments

Line 95-96

I find it imprecise to say that isotopes are incorporated into animal tissues through trophic level interactions. You might more closely reflect the relationship of animal tissues to isotopes by stating that they are incorporated through biochemical processes

(e.g. Breathing is, in part, responsible for the isotope ratios in an organism’s tissues).

Line 103-104

As with my comment on Line 95, I suggest that the δ2H isotope ratios in animal tissues reflect their environment not singularly because of trophic interactions.

You might also reconsider the use of the word “highly” to describe the relationship between tissues and δ2H isotope ratios, given the many confounding factors that obscure this relationship (e.g. evapotranspiration of environmental or biological water differs among individuals and perhaps small-scale location). I think the theme of quantifying the factors that obscure δ2H_P_ and δ2H in animal tissues, in particular, is common in recent papers.

You might also consider the addition “expected to reflect the isotopic signatures of the elevation of feeding *when the tissue was grown*”

Line 157

I’d suggest that “greater inter-tissue *variation*” might better capture your meaning here.

Line 189

Were tail feather selected for any reason? The outer tail feathers are molted last in many species. Is there a possibility to mis-classify some lower-elevation migrants as higher-elevation locals by sampling feathers that were recently replaced (i.e. outer tail feathers, the last replaced) at higher elevations? If this went unaccounted for, you might actually find stronger support for your elevational-migrant hypothesis.

Further, were the tail feathers counted from the center to determine the feather taken was the true outer feather and not the outermost that is not actively being replaced? Failure to account for this could introduce additional unwanted noise into your data.

Lines 266-267.

The cutoff of 2‰ used to assert that differences between ‘apapane tissues owe to differential discrimination while in differences between tissues amakihi’ are due to diet changes seems arbitrary. Without stronger *apriori* assertions about 2‰ it seems equally valid to conclude that amakihi’ demonstrated a greater seasonal shift in diet than ‘apapane. Or that the isotope discrimination between the two tissues was greater in ‘apapane than amakihi’. Further, I’d suggest this is best left for the discussion rather than the results section.

Line 272-274

Does the larger variation in δ13C not also suggest a more diverse diet? Regardless, this sentence might be best positioned in your discussion section.

Lines 352-355

I think this is an important distinction for your study area. Good inclusion.

General Comments

A) I assume all birds used in your study were AHY individuals? Is this simple to determine using plumage differences between AHY and HY? Other studies have found significant isotope ratio differences between AHY and HY individuals at the same breeding site. (Hache et al. 2012 - Assigning birds to geographic origin using feather hydrogen isotope ratios (δ2H): importance of year, age, and habitat)
B) **The most important concern with the manuscript**: I am suspicious of the application of a conversion equation that describes the relationship between δ2H_P_ and δ2H_f_ in hummingbirds to your study species. Elsewhere, among closely related species breeding in the same area, species-specific equations to convert δ2H_P_ to δ2H_f_ appear to be necessary (Nordell et al. 2016 - Within-Site Variation in Feather Stable Hydrogen Isotope (δ2 Hf) Values of Boreal Songbirds: Implications for Assignment to Molt Origin).

I am no biochemist, but the particularly extreme metabolism of hummingbirds might lead to biochemical isotope discrimination at significantly greater rates than perhaps any other species.

I am not sure whether the fast hummingbird metabolism would incorporate light hydrogen or deuterium (heavy hydrogen) preferentially in metabolic processes. The worst case scenario for your manuscript would be that deuterium is more concentrated in hummingbird tissues than in your study species, which would lead you to predict δ2H values in your species originated at lower elevations than they truly did. In that case, you might find considerably less support for your elevation-migrant hypothesis.

I suggest you explore the literature to determine whether the use of the conversion equation for hummingbirds is valid (I don’t think the fact that they are nectivores is justification enough) and, if necessary, utilize a conversion equation more representative of your species (e.g. other fringilidae).
